# Supplementary material for: Transport and Electrochemical Characteristics of CJMCED Homogeneous Cation Exchange Membranes in Sodium Chloride, Calcium Chloride, and Sodium Sulfate Solutions
Source: Membranes (Basel). 2020 Jul 25;10(8):165. doi: 10.3390/membranes10080165 (PMC7463934; doi:10.3390/membranes10080165)
Supplement: Supplementary file 1 [file membranes-10-00165-s001.pdf]

Supplementary materials

Chemicals

Table S1. Some characteristics (at 25°C) of ions included in the studied solutions. The data are borrowed from [73].

Ion	Crystallographic radius, Å	Stokes radius, Å	Hydration energy, kJ mol^{-1}	Hydration number	Diffusion coefficient at infinite dilution $D_0 \times 10^9$, m^2 s^{-1}
Na^+	0.95	1.84	407	5	1.334
Ca^{2+}	0.99	3.10	1584	9	0.792
Cl^-	1.81	1.21	376	2	2.032
SO_4^{2-}	2.90	2.30	1138	14	1.065

Methods

The total exchange capacity (Q_{sw}) of studied CEM under study is determined by the static method [72]. A sample of the swollen membrane (about 1.0 g (m_{sw})), was initially transformed into the H^+ form by soaking in a 1M HCl solution. Then, after careful rinsing in deionized water, the sample was immersed in a 20.0 cm^3 of a 0.1M NaCl solution to replace H^+ ions by Na^+; it was kept there until equilibrium (for 24 hours). After that, the concentration of the H^+ ions released into the solution was determined using the potentiometric titration with a 0.1 M NaOH. The titration was performed using EasyPlusTitrators (METTLER TOLEDO), with the output of the titration results to a computer.

The calculation of the membrane exchange capacity per weight of swollen membrane, Q_{sw} (mmol g_{sw}^{-1}), was carried out by equation:

$$Q_{sw} = \frac{V_T c_T}{m_{sw}} \quad \text{(S1)}$$

where V_T is the volume of 0.1 M NaOH solution, spent on titration (mL), c_T= 0.1 mmol/mL (NaOH); m_{sw} is the mass of swollen sample (g).

Water content (W, %) of the membranes is determined by the gravimetric method. Before the experiment, the samples were equilibrated with 0.02 mol dm^{-3} electrolyte solution at 25 ± 1 °C for 24 h. After equilibration, samples were taken out from the solution and the film of liquid was removed from the samples' ends and surfaces using filter paper.

Weights of wet, m_{sw}, and dry, m_{dry}, samples were obtained using an MB25 Ohaus moisture analyzer. The evaporation of water was carried out at a temperature of 50 °C to a constant weight of the sample.

The water content W, % was calculated by the formula:

$$W = \frac{m_{sw} - m_{dry}}{m_{dry}} \times 100\% \quad \text{(S2)}$$

Electrical conductivity of IEMs (κ^*) was determined by a differential method using a clip cell [56, 59] and a АКИП 6104 immitance meter (Motech Industries Inc., Taiwan) at an AC frequency of 1 kHz. All samples were studied in 0.02–1.00 eq L^{-1} solutions of NaCl, $CaCl_2$, and Na_2SO_4, starting from the lowest concentration.

The conductivity of the membranes (κ^*) was found by the formula:

$$\kappa^* = \frac{d_m}{R_{m+s} - R_s} \quad \text{(S3)}$$

where R_{m+s} is the resistance of the membrane in solution and R_s is the resistance of the solution alone. The obtained concentration dependences of electrical conductivity were processed using the microheterogeneous model [61, 71] to determine the volume fractions of the gel phase (f_1) and the phase of the electroneutral solution that fills the intergel spaces (f_2) of the investigated membranes and to estimate the electrical conductivity of the gel phase $\bar{\kappa}$:

$$\kappa^* = \bar{\kappa}^{f_1}\kappa^{f_2} \quad \text{(S4)}$$

where κ is the specific electrical conductivity of the "intergel" solution, assumed to be equal to that of the external equilibrium solution.

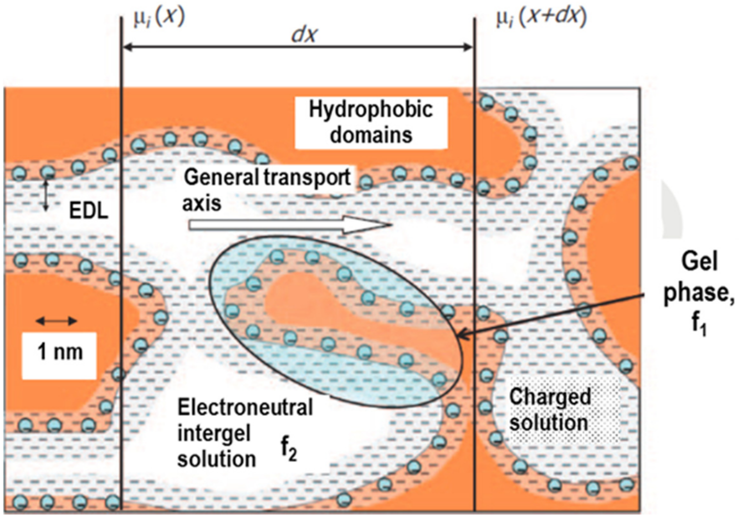

Figure S1. Cross-section of the ion exchange membrane bulk; a scheme illustrating the microheterogeneous model [61]

According to the microheterogeneous model, an IEM is considered as a two-phase system ($f_1 + f_2 = 1$) in the simplest case. The gel phase is a microporous swollen medium (**Figure S1**). It includes the polymer matrix, which bears charged fixed groups, and the charged solution of mobile counterions and, in a smaller number, coions that compensate for the charge of fixed groups. The reinforcing cloth fibers and the inert filler (polyethylene) are also included in the gel phase. The second phase is formed by an electrically neutral solution (identical to the external solution) that fills the intergel spaces. This solution includes the fluid in the central part of the meso- and macropores and in the structural defects of the membrane. In the first approximation (when the presence of coions in the gel phase is neglected), the electrical conductivity of this phase is considered to be constant, depending on the counterion diffusion coefficient in the gel phase of the membrane, $\bar{D}_i$, and on its exchange capacity, $\bar{Q}$:

$$\overline{\kappa} = \frac{z_i \overline{D_i Q} F^2}{RT} \quad \text{(S5)}$$

where F is the Faraday constant, R is the universal gas constant, T is the temperature, and z_i is the counterion charge. The value is related to the ion-exchange capacity Q of the membrane by $\bar{Q} = Q/f_1$. The numerical value of $\overline{\kappa}$ can be determined from the value of the membrane electrical conductivity (κ^*) at the isoconductivity point in which the conductivities of the membrane (κ^*) and the solution (κ) are identical. It is clear that according to Equation (S5), the following equality holds in this case: $\kappa^* = \kappa = \overline{\kappa}$. For commercial membranes and strong electrolytes, the concentration at this point (C_{iso}) is not far from 0.05 mol L^{-1} solution (**Figure S2a**): at $C < C_{iso}$, the conductivity of the membrane is higher than that of the solution, $\kappa^* < \kappa$, and at $C > C_{iso}$, $\kappa^* > \kappa$. According to Equation (S4), the $lg\kappa^*$ vs. lg κ dependence gives a straight line, which is well confirmed by experimental data (**Figure S2b**). The slope of the line is equal to f_2 (if $\overline{\kappa}$ is assumed constant). The free term b of the equation $y = ax + b$ approximating the trend line of the dependence $lg\kappa^*$ vs. $lg\kappa$ is equal to $f_1 lg\bar{\kappa}$.

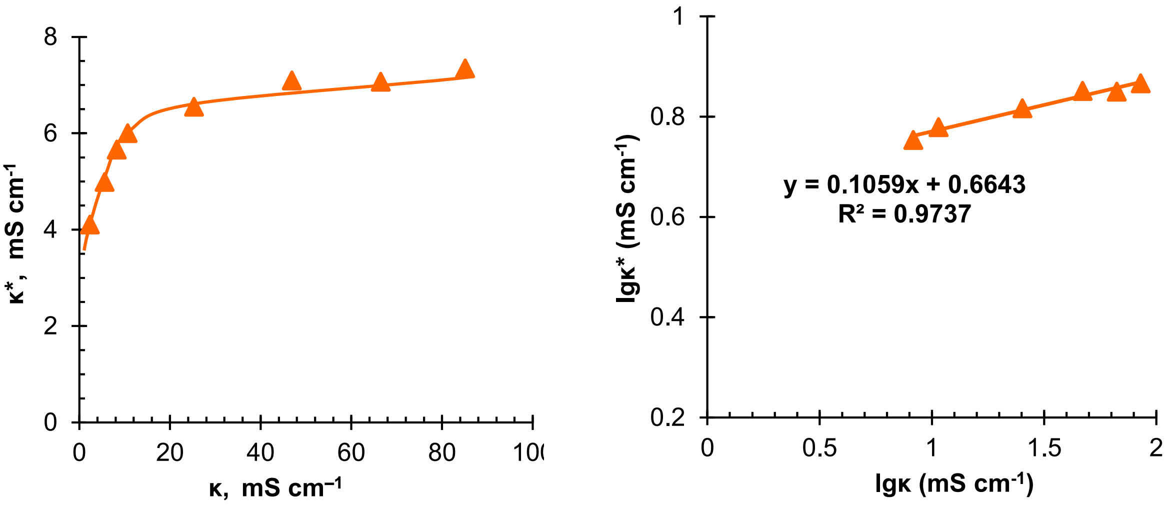

(**a**) (**b**)

Figure S2. Concentration dependences of the specific electrical conductivity of CMX membrane (κ^*) upon the specific electrical conductivity of NaCl solutions (**a**) and $lg\kappa^*$ vs. $lg\kappa$ (**b**) coordinates. The line in (**a**) is drawn to guide the eye; the straight line in (**b**) is the linear trendline

The diffusion characteristics of IEMs were studied using two-compartment flow-through cell *1* [62] equipped with special devices for the inlet and outlet of the solution (**Figure S3**). These devices provide the laminar flow of the solution in cell compartments *3* and *4*, which makes it possible to use the Leveque equation for calculating the average thickness of diffusion layers, δ, near the surface of the IEM under study [61]:

$$\delta = 1.02(LDh/\bar{V})^{1/3} \quad \text{(S6)}$$

where L is the length of the channel, cm;   is the distance between test membrane *2* and the cell wall, cm; D i is the diffusion coefficient of the electrolyte, cm²/s; and $\overline{V}$ is the average linear flow velocity of the solution, cm s^{-1}. The values of   were 0.63 cm, and the working area of the membranes S was 2.7×2.7 cm².

The membrane under study, which was preliminarily equilibrated with a 0.1 M electrolyte solution (NaCl, $CaCl_2$ or Na_2SO_4) was placed into cell *1*. Prior to each experiment, 1000 cm³ of the electrolyte solution with a set concentration was poured into flowing tank *6* and compartment *4* (stream II). Distilled water in an amount of 100 cm³ was poured into flowing tank *5* and compartment *3* (stream I). In 40 min after starting the experiment, the solution from this duct was rapidly drained, and the tank was refilled with distilled water. The average linear velocity of the solutions circulating through each of the cell compartments was 0.90±0.10 cm s^{-1}. Every 30 s, the values of the electric conductivity and pH of the solution in container *5* were recorded using immersion conductivity cell *9* and combined glass electrode *15* connected to an Expert 001 conductometer and an Expert 002 pH meter.

The values of the integral diffusion permeability coefficient were calculated according to the known equation [71]

$$P = \frac{d_m V_I}{SC_{II}} \frac{dC_I}{dt}, \quad \text{(S7)}$$

where d_m is the thickness, S is the area of the membrane under study, V_1 is the volume of the solution in tank I, C_{II} is the concentration of the solution in tank II, and dC_1/dt is the rate of concentration growth in stream I. The latter is determined using the regression analysis of the linear portion of the time-dependence curve of the electric conductivity of the solution in container *5* (initially filled with pure water). The studies were performed at different concentrations of NaCl in stream II.

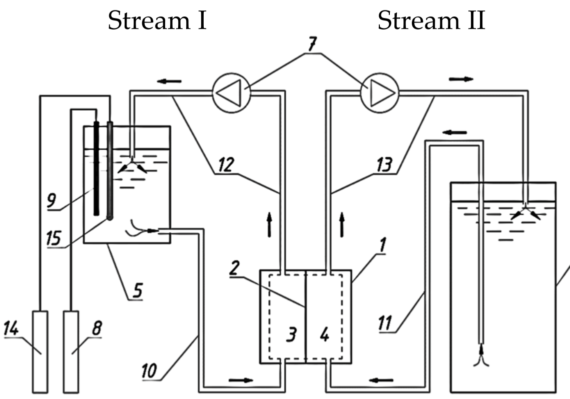

Figure S3. Schematic of the unit for measuring the diffusion permeability of membranes: (1) two-compartment cell, (2) membrane under study, (3, 4) flow-through compartments of cell 1, (5) tank with distilled water, (6) tank with an electrolyte solution of the set concentration, (7) pumps, (8) conductometer, (9) immersion conductometric cell, (10–13) connecting hoses, (14) pH meter, and (15) combined glass electrode for pH measurements.

In the framework of the microheterogeneous model [61, 71, 73], the differential coefficient of diffusion permeability P^* is determined by the diffusion permeability of the membrane gel phase, $\bar{P}$, and the diffusion coefficient of the electrolyte, D, in the solution located in the intergel spaces:

$$P^* = \left\{\left[f_1\left(\frac{\bar{P}}{\bar{t_1}}\right)^{\alpha} + f_2\left(\frac{D}{t_1}\right)^{\alpha}\right]^{-1/\alpha} + \left[f_1\left(\frac{\bar{P}}{\bar{t_A}}\right)^{\alpha} + f_2\left(\frac{D}{t_A}\right)^{\alpha}\right]^{-1/\alpha}\right\}^{-1}, \quad \text{(S8)}$$

where α is a structural parameter. Its value depends on the disposition of the phases (domains) relative to the transport axis. $\bar{t_i}$ and t_i are the transport numbers of ions i in the gel phase and in the electrolyte solution, respectively. The subscript i has the values *1* (counterion) and *A* (coion).

The diffusion permeability of the gel phase, $\bar{P}$, is mainly determined by the concentration $\bar{c}_A$ and diffusion coefficient $\bar{D}_A$ of coions in this phase [73]:

$$\bar{P} = \left(1 - \frac{z_A}{z_1}\right)\bar{t_1}\frac{\bar{D}_A\bar{c}_A}{c_A}, \quad \text{(S9)}$$

where z_1 and z_A are the charge numbers of counterions and coions, respectively (the sign of the charge is considered), c_A – is the concentration of coions in the external solution. The value of $\bar{t_1}$ differs little from 1 in a wide range of concentrations. Therefore, coions make the decisive contribution to the value of $\bar{P}$. Theoretical estimates [72, 73, 70] and experimental results [62] allow concluding that $\bar{c}_A/c_A$ <<1, and $\bar{D}_A$ is one or two orders of magnitude smaller than the diffusion coefficient in the external solution. With increasing f_2 the volume fraction of the solution, which is equivalent to the external solution, increases. Thus, the diffusion permeability of membranes, whose f_2 is higher than about 0.1, increases.

To assess the effect of the external solution concentration, the ion-exchange capacity of the membrane and the charges of coions and counterions on the diffusion permeability of the membrane, it is convenient to use the following equation [73]:

$$\frac{\bar{c}_A}{c_A} = K_D^{|z_A|}\left(\frac{c}{\bar{Q}}\right)^{|z_A/z_1|}, \quad \text{(S8)}$$

where K_D is the Donnan constant. This equation is deduced from the Donnan relation considering that the concentration of coions in the gel phase of the membrane is much lower than the ion-exchange capacity $\bar{c}_A \ll \bar{Q}$ ($\bar{Q} = Q/f_1$). This equation holds in relatively dilute solutions.

According to Equation (S8), for a given value of $\bar{Q}$, the concentration of coions in the gel phase decreases with dilution of the solution. A decrease in this concentration leads to a decrease in the diffusion permeability of the membranes.

Another parameter that affects the diffusion permeability of the gel phase is the ratio $|z_A/z_1|$. Indeed, substituting Equation (S8) in Equation (S7) yields:

$$\bar{P} = \left(1 - \frac{z_A}{z_1}\right)\bar{t_1}\bar{D}_A K_D^{|z_A|}\left(\frac{c}{\bar{Q}}\right)^{|z_A/z_1|} \quad \text{(S9)}$$

or

$$\bar{P} = 2\bar{t}_1\bar{D}_A K_D\left(\frac{c}{\bar{Q}}\right), \qquad |z_1| = |z_A| = 1 \tag{S10}$$

$$\bar{P} = \frac{3}{2}\bar{t}_1\bar{D}_A K_D\left(\frac{c}{\bar{Q}}\right)^{1/2}, \qquad |z_1| = 2, |z_A| = 1 \tag{S11}$$

If the values $\left(1-\frac{z_A}{z_1}\right)\bar{t}_1\bar{D}_A K_D^{|z_A|}$ are close for the studied electrolytes, and$c/\bar{Q} \ll 1$, then $\bar{P}_{|z_1|=|z_A|=1} < \bar{P}_{|z_1|=2,|z_A|=1}$.

The current-voltage characteristics (CVC) are measured in galvanodynamic mode at a current sweep rate of 0.02 mA s^{-1}. Along with CVC, pH is recorded at the inlet and outlet of the DC, which is formed by the membrane under study and an auxiliary membrane (**Figure S4**). The volume of NaCl (0.02 M, pH 5.8 ± 0.2) solution poured into tank 1 before the experiment, is 5 dm^3. This solution is supplied to all the compartments of the cell and then return to the same tank. Due to the relatively large volume of the solution circulating through tank 1, the deviations of the concentration of its components from the initial value during one run of the experiment is not exceed 1%.

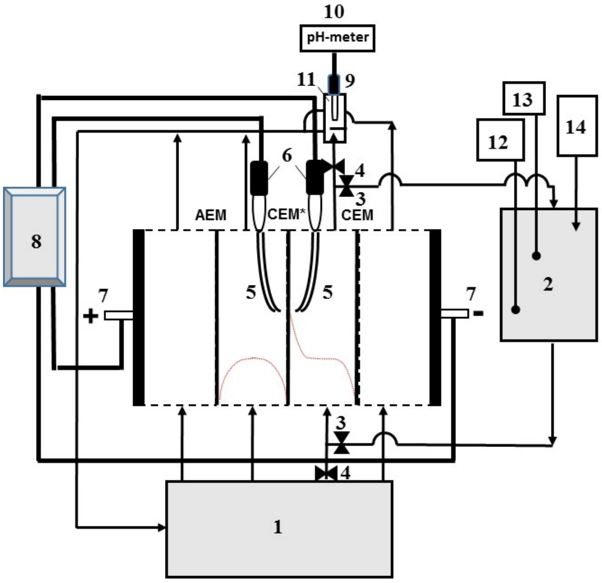

Figure S4. Schematic design of the set-up used for determining mass transfer and electrochemical characteristics of the CEM membranes forming the desalination compartment. The set up includes: an intermediate feed tank (1); an additional tank (2) for maintaining a constant pH; valves (3, 4); Luggin capillaries (5) connected with measuring Ag/AgCl electrodes (6); platinum polarizing electrodes (7); an electrochemical complex (an Autolab PGSTAT-100) (8); a flow-through cell (9) with an immersed combination electrode (11) for pH measurement; a pH meter (10) connected to a computer; a conductivity cell (12) connected to a conductometer; a device (13) for maintaining a constant pH in tank (2), which is controlled with a combination electrode (14); CEM* is the cation-exchange membrane under study (CMX, CJMC – 5 or CJMC - 3); CEM and AEM are the auxiliary cation-exchange and anion-exchange membranes needed to avoid the transfer of ions H^+ and OH^- from the electrode compartments to the compartments next to the studied membrane. The dotted lines schematically show the electrolyte concentration profiles in the compartments next to studied membrane.

The potential drop measured between the Luggin capillaries (5) depends on the distance between the membrane and capillaries (5), the thickness of the membrane and diffusion layer and the conductivity of the membrane and solution [98]. These parameters are difficult to find and reproduce when replacing one membrane with another one. To exclude this difficulty, the corrected potential drop $\Delta\varphi'$ is used instead of the total potential drop $\Delta\varphi$ [66]:

$$\Delta\varphi' = \Delta\varphi - iR_{ef} \tag{S12}$$

where the effective resistance of the membrane system R_{ef} (Ohm cm^2) includes the ohmic resistance of the space (membrane + solution) between the measuring capillaries, as well as the diffusion resistance of the interphase boundaries of depleted and enriched diffusion layers [54]. The value R_{ef} is found from the initial part of CVC by extrapolation $i \to 0$ in the coordinates $i - d\varphi/di$.

The limiting current density is calculated using the Leveque equation obtained in the framework of the convective-diffusion model [67]. It can be expressed as:

$$i_{lim}^{Lev} = 1.47\frac{FDz_1C_1}{h(1-t_1)}\left(\frac{h^2V}{LD}\right)^{1/3}, \tag{S13}$$

where F is the Faraday constant; D is the electrolyte diffusion coefficient; z_1 is the charge number of the counterion; C_1 is the molar concentration of the counterion in the solution at the entrance to the desalination compartment; h is the intermembrane distance; t_1 is the electromigration transport number of the counterion in the solution; V is the average linear flow velocity of the solution; L is the length of the membrane working area.

The thickness of the diffusion layer, δ, is estimated by combining the Leveque and the Peers equations:

$$\delta = 0.68h\left(\frac{LD}{h^2V}\right)^{1/3} \tag{S14}$$

The calculations are made with t_i equal to 0.396 (Na^+) and 0.604 (Cl^-); D = 1.61×10^{-9} m^2s^{-1}, which is an value for the diffusion coefficients of NaCl electrolyte with infinite dilution [55]. The values for i_{lim}^{theor} for the system under study equal to 3.0 mA cm^{-2} (AEM) and 2.0 mA cm^{-2} (CEM). The thickness of the diffusion layer, δ, in the DC formed by these membranes is 250 μm according to Equation (S14).
